# Contesting the deportation state? Political change aspirations in protests against forced returns

**DOI:** 10.1080/01419870.2018.1562194

**Published:** 2019-02-01

**Authors:** Leila Hadj Abdou, Sieglinde Rosenberger

**Affiliations:** a Migration Policy Centre, Robert Schuman Centre for Advanced Studies, European University Institute, Florence, Italy; b Department of Political Science, University of Vienna, Vienna, Austria

**Keywords:** Asylum, immigration, deportation, protest, political change, Austria

## Abstract

Deportation of immigrants is a high-ranking issue on political agendas across Europe. Political authorities, however, face a challenge regarding forced returns: affected migrants, organized activists and concerned citizens are standing up for deportees. Do these protests contest the nation state’s sovereignty, expressed in the right to carry out deportations of foreign citizens? How far-reaching are protesters’ ambitions for political changes? Based on a developed typology of change aspirations, this article explores this topic by studying anti-deportation protests in Austria. It combines qualitative data from interviews with protesters with longitudinal data covering protest events from 1993 to 2013. Expanding previous research, the study finds that protests often refrain from demanding fundamental political change, instead they demonstrate overt conformism for tactical purposes. At the same time, protesters develop grievances about deportation policies and practices in the course of protest developments – they have covert reformist ambitions.

## Introduction

Strengthening national sovereignty and taking back control over national borders have become rising political imperatives across Western democracies. The forced removal of unauthorized residents plays an important role in this political agenda. The political commitment to deportation is often pursued in the name of “the people”, as a response to citizens’ discontent with irregular immigration (Drotbohm and Hasselberg 2015, 565; Wong 2015). However, deportations do not go unchallenged. On the contrary, there have been protests staged by advocacy groups, affected migrants, organized activists and by concerned citizens. Instances of case mobilization in particular, i.e. cases in which people have protested against the enforcement of deportations in solidarity with specific immigrants, have gained much public attention and visibility (Buff 2018; Ellermann 2009; Freedman 2011).

Anderson, Gibney, and Paoletti (2011) have argued that case mobilizations are a two-edged phenomenon in terms of questioning deportation. While these struggles expand the boundaries of community by including some individuals who have no legal right of residence, there is less evidence that those who engage in solidarity movements for individuals challenge the practice of deportation i.e. the right to remove foreign citizens from national territory, as such (Anderson, Gibney, and Paoletti 2011, 559–560). In line with such findings, other studies have argued that protests for specific deportable individuals neither question the restrictive regulation of migration, legal status and residence rights nor the underlying fundamentals that curtail the right to movement of the many “unwanted” aliens while upholding the privilege of a freedom to migrate for the few (Peutz and De Genova 2010). Bader and Probst (2018) studying Switzerland even note that protesters engaging against the enforcement of individual deportations stand together precisely because these advocacy groups do not carry any absolute claim to freedom of movement and they do not express a desire for transformation of the social and/or political order. Studies (Ellermann 2006; Patler and Gonzales 2015; Rosenberger and Winkler 2014) stress that in case mobilizations the topos of deservingness is often a core campaign element. The right to stay is not claimed an absolute but as a conditional right. In a conformist way, protesters demand that certain individuals should be allowed to stay as a legitimate exception to the rule; an exception for a few who are deemed worthy.

The article takes these scholarly discussions about demands and underlying aspirations for change in protests for individuals as a starting point. While providing important reflections on the political goals of anti-deportation protests, research to date has not paid much attention to conceptualizing and systematizing political change aspirations, nor has it provided adequate answers to the question of how to understand the limited aspiration for change in case mobilizations. Against this briefly sketched theoretical and empirical background, this paper asks whether case mobilizations are primarily about saving certain individuals or about tackling the wider deportation regime. In particular, the paper is interested in answers to the question of how to understand rather modest change aspirations concerning the statecraft of deportations and ways of enforcing them. Thus, this article aims to contribute to a scholarly discussion about different scopes of political change aspirations within anti-deportation protests and to deepening the understanding of moderate change aspirations within case mobilization.

Interested in a fine-grained analysis of change aspirations, we developed a three-dimensional typology of anti-deportation protests. Based on theoretical knowledge on collective mobilization and on an inductive approach to the media material we draw on in this paper, we conceptualize change aspirations covering a transformational/radical, reformist/substantive, and conformist/moderate dimension. We will apply this typology to systematize the claims identified in media reports in the field of deportation.

Empirically, we will investigate this topic focusing on protests in Austria. The article draws on original empirical data we collected within a larger, comparative research project on anti-deportation protests in three countries (Austria, Switzerland, Germany). Findings of the project (see Ruedin, Rosenberger, and Merhaut 2018) have shown that anti-deportation protests in Austria have been predominantly protests for individual deportees. While comparative findings of the research project on manifestations of deportation protests are presented elsewhere (Ruedin, Rosenberger, and Merhaut 2018), in this article we deliberately focus in depth on a single country. This allows us to contribute substantively to the research on political change aspirations in case mobilization. The analysis presented in this article uses a triangulation approach, combining data from quantitative protest event analysis over twenty years with qualitative interviews with protest actors.

Our analysis reveals that the extent to which anti-deportation protests in Austria openly challenge power holders and deportations as the “standardized instrument of statecraft” (Peutz and De Genova 2010, 3) is rather limited. Protests tend to refrain from contesting the practice of deportation, calls for policy reforms are secondary. This overall conformist character of protests is to be understood as a deliberate tactic, which is used by protesters to make themselves heard in an anti-immigrant environment and to achieve the primary goal of protecting specific individuals from deportation. Although only moderate change aspirations are exhibited on the surface, the conformist pattern does not necessarily mean that protesters do not hold grievances about the deportation system, policies and enforcement practices. As the findings presented below underline, in the course of mobilization for individuals, compassion for a few can turn into substantive discontent about laws and policies.

After an introduction, the article is divided into six sections. We start by describing the case of Austria in terms of protest culture and attitudes to immigration. We then provide details on the material and methods used. In the third section, a typology of political change aspirations in anti-deportation protests is presented, followed by empirical findings for Austria (fourth section). Section five explores the drivers of the dominant political change aspiration identified in the Austrian case. In the sixth section, we summarize our findings.

## Austria: strong contestation of immigration, but weak protest culture

Austria, for several reasons, is a particularly interesting case for exploring political change aspirations in protests in the field of migration. First, despite it being a country with a long history of immigration and a significant number of foreign nationals, Austria’s political elites have been reluctant to define Austria as a country of immigration. Since the early 1990s, immigration policies have been guided by an “unspoken desire for a new Iron Curtain” (Fassmann and Münz 1995, 91). Comparative research has demonstrated that the immigration issue has been more salient in Austria than in most other European immigration countries (Meyer and Rosenberger 2015, 30). Public opinion towards immigrants has been relatively hostile and a high proportion of the population are in favour of restrictive immigration policies (Meyer and Rosenberger 2015, 32). According to Eurobarometer data of spring 2018, 57 per cent of the Austrian population see immigration from outside the European Union as a negative phenomenon. At the peak of the 2015 refugee crisis, the number of people holding negative views towards third-country nationals was even as high as 64 per cent (MIDEM 2018, 127). Moreover, the Austrian Freedom Party (FPÖ) is known across Europe as a role model for the populist radical right and has successfully mobilized the anti-immigration vote. In this context, immigration has been a very contested issue for decades. Given the salience of the issue and the strength of the populist radical right, Austria has been characterized as an anti-immigrant “heartland” (Bale and Gruber 2014). This character is mirrored in restrictive immigration control and asylum policies, and a strong deportation machinery (Horvath 2014).

Secondly, Austria has a comparatively weak protest culture from below (Hutter and Teune 2012, 9). This general pattern also applies to the issue of immigration. Mass mobilizations in the field of immigration are rare (Bader and Probst 2018). Politicization of immigration is, thus, mostly a top-down phenomenon. Interestingly, the issue of the deportation of foreign citizens with no appropriate legal residence status, predominantly of rejected asylum seekers, is to some extent an exception to the pattern of a feeble protest culture. Even under unfavourable political circumstances, anti-deportation protests have occurred since the early 1990s. For the time period studied (1993–2013), protest activities reported in the media have even increased although the number of deportations has decreased over the same period. The issue of deportation has become politicized by non-institutional actors over time. Moreover, anti-deportation protests have been effective in preventing enforcement. According to empirical studies, between 2006 and 2013, in almost two-thirds of cases, altruistic protests succeeded in preventing the enforcement of a deportation order (Rosenberger and Winkler 2014).

Before we turn to our findings from the Austrian case, we now describe the material and methods used.

## Material and methods

This article draws on a triangulation of different data sets, which we^1^ collected for a comparative research project (“Taking Sides”) on features and conditions of anti-deportation protests in three countries (Austria, Germany and Switzerland). In this research project, we combined quantitative data collection with qualitative research. First, the research project undertook a protest event analysis (PEA), capturing all anti-deportation protest events reported in print media in the three countries between 1993 and 2013. The period chosen for this part of the study is one in which immigration control rose high on the political agenda in Western Europe, Austria included. Secondly, five case studies on major protest cases that happened between 2005 and 2015 were conducted based on semi-structured interviews with protest actors.

For the PEA, articles on deportations published in major national newspapers were used (for Austria: Die Presse, Der Standard). In response to the media environment of the past decade, protest research has increasingly turned from using quality newspapers to using online sources (Kousis, Giugni, and Lahusen 2018, 741). However, given that this study covers anti-deportation protests back to the early 1990s, an analysis of quality print media was carried out. Protests featured in the print media are particularly powerful in triggering responses on the part of policy makers (Van der Brug et al. 2015). Based on key word searches, 311 deportation protest events were identified and manually coded using the AmCat software to examine project trajectories, protest frequency, main actors, repertoires, claims and frames. The codes were developed based on an inductive approach and refined in the course of research. In this article, we will mostly refer to findings on claims, i.e. the demands brought forward by protest actors. When coding claims, we looked for the primary claim for each protest event. This coding strategy allowed us to identify protest claims in a systematic fashion, distinguishing between nine different types of claims (see Table 2 for a list of types of claims). To illustrate the coding process: “No one is illegal. Stop deportations” would be coded as a claim directed against the deportation system. Statements like “Stop deportations to Greece”, or “Prohibit the deportation of minors” are examples of claims we would code as demands for a reform of policies or enforcement practices. “Do not deport our classmate [Name]” would be coded as a moderate claim to prevent the deportation of a specific individual.

For the protest case studies, each case consisted of a series of events, which emerged in the context of the enforcement of specific deportations.^2^ All the cases sparked a significant public debate, and so qualify as key cases. For the selected five cases, 27 semi-structured interviews with protest actors were conducted in 2015. The interviews were conducted in German and translated into English. Only actors who had protested on behalf of migrants were interviewed. We analysed these interview data by applying a summative content analysis (Hsieh and Shannon 2005, 1283–1285). For ethical reasons, we anonymized all quotations from the interviews used in this article.

## An analytical framework: change aspirations in anti-deportation protests

To identify, classify and understand different scopes of change aspirations in anti-deportation protests, we will first turn to a brief discussion on the central meaning of deportation within the nation state, as well as on the relationship of protest with political/policy change. Based on this discussion, we will then present the developed typology for political change aspirations in anti-deportation protests which we developed and which provides the conceptual scheme for our analysis.

Deportations are discussed as a manifestation of the right of sovereign states to control the entry and stay of foreign citizens on their territory. In line with this perspective, the practice of deportations can be viewed as a “purely administrative corrective” of removing people that are “out of place” (De Genova 2014). From a legal perspective, deportations are compatible with the liberal principles of modern nation states and they are widely perceived as legitimate for protecting national territory and host communities, as well as ensuring the integrity of asylum systems for refugee protection (Gibney 2013, 123). At the same time, such a perspective, as critical scholarship on deportation and citizenship has pointed out (e.g. Aas and Bosworth 2013; Peutz and De Genova 2010), ignores processes of normalization and routinization of deportation, as well as inequalities and unequal power relations that are (re)produced by immigration control regulations and practices. At the beginning of the twentieth century, as Peutz and De Genova (2010, 24) emphasizes, it was commonly considered unconscionable, even by some immigration judges, to deport irregular but otherwise lawful long-term residents and their families. At the end of the century, it had become “utterly banal” (Peutz and De Genova 2010). Deportation is constitutive for the foundations of modern society, it (re)produces the formal and normative boundaries of national citizenship and reaffirms the differential treatment of foreign citizens (see Anderson, Gibney, and Paoletti 2011; Drotbohm and Hasselberg 2015, 552; Walters 2002). This differential treatment is consequential. It grants citizens, as opposed to aliens, advantages on the basis on birth and entrenches these birth privileges by legally restricting the movement of people (Carens 1987). Immigration and citizenship regimes also entail hierarchies of national exclusion that privilege some forms of mobility over others (cf. Anderson, Sharma and Wright 2009, 10). Put crudely, immigration control is fundamentally about regulating North-South relationships and maintaining inequalities, as Castles (2004; cit. after Castles 2017, 1542) has argued.

Anti-deportation protests thus target a policy area which lies at the very heart of the nation state and the constitution of (stratified) boundaries of the national political community. Protests against deportation consequently question the boundaries between exclusion and inclusion and, in doing so, disrupt, albeit to differing degrees (depending on which political change they stand up for), “defined orders, practices and statuses” (Isin 2009, 384).

With regard to the relationship between protest and change aspirations, social movement scholarship has tended to focus on large-scale phenomena, such as the civil rights movement, that can be classified as a sustained challenge to power holders and a demand for radical changes in political actors, political processes and policies. In doing so, social movement studies have made an invaluable contribution to our understanding of mobilization and contentious politics. Less radical demands, however, are harder to fit within the current instruments employed in social movement research. In response, Diani and Bison (2004, 285) have developed the concept of consensus mobilization, i.e. types of mobilization in which envisaged solutions to perceived grievances do not necessarily require redistribution of power or alterations in socio-political structure, as opposed to types of mobilization that imply more far-reaching changes and power struggles.

More broadly spoken, protest is about producing, halting or reversing change (Schaeffer 2014, 11). However, the scope of change aimed at through protest can differ markedly. Some protests may call for the transformation of the very foundations of society and politics. Others focus on less far-reaching objectives, such as a legal reform, or a change in administrative practices (Edwards 2014, 4). These protests seek to correct problems in the social and/or political order, but they leave the underlying structures intact. Finally, some protests do not touch upon the social and/or political order at all: they aim neither to transform nor to correct it (Chimienti and Solomos 2011, 350), envisaged change might be limited to finding individual, or one-off solutions to address grievances.

Based on the protest literature as well as on deportation, immigration and citizenship studies referred to above, we distinguishing between three different types of change aspirations that can occur in anti-deportation protests (see Table 1).

**Table 1.** Typology of change aspirations in anti-deportation protests.

	Transformational/radical	Reformist/substantive	Conformist/moderate
Dimension	Political/social order	Legislative regulations	Case enforcement
Problem definition	The right of nation states to restrict the freedom of movement of foreign citizens is unjust	Regulations and/or implementation are too restrictive	Costs of exclusion for individual deportees and/or society are too high
Problem solution	Abolishing nation states’ sovereignty as regards the regulation of mobility	Reform of policies and/or policy implementation on residence and return	Preventing the deportation of individuals
Expression	Claims against restrictions of movement	Claims for liberalizing policies/implementation	Claims against deportations in individual cases

Source: Authors’ illustration.

### Transformational change aspirations

We speak of transformational (or radical) change aspirations when protesters target the social and political order on which deportations are based. Transformational protest claims would contain a rejection of the statecraft of deportation as such. This change aspiration is seen as radical as it provokes the power of sovereign nation states to admit or exclude aliens, i.e. it basically questions the idea that movement by individuals should be managed and restricted by nation states. We thus classify change aspirations as radical if they express claims directed against deportation as a legitimate fundamental practice of nation states (Chimienti and Solomos 2011, 350).

### Reformist change aspirations

Claims containing reformist change aspirations do not tackle the foundations of the practice of deportation, they do not question the right of sovereign nation states to exclude aliens, but aim to reduce the scope of exclusion by liberalizing asylum and immigration regulations and enforcement practices. In other words, protest claims of a reformist character are about rendering the juridical framework more inclusive, e.g. by loosening conditions for entry and residence, while keeping existing arrangements intact. They call for fairer immigration and asylum laws (Anderson, Sharma, and Wright 2012, 82) and/or fairer ways of enforcing the law. We identify change aspirations as reformist if they carry claims for policy reform, or for a reform of deportation enforcement practices.

### Conformist/moderate change aspirations

In conformist aspirations for change, claims are restricted to tackling exclusion in specific instances. These protests target the enforcement of deportations in individual cases for reasons grounded in personal characteristics of these individuals. They do not question the general idea that aliens are deportable subjects based on citizenship, residence and migration laws. Nor do they aim to reduce the number of deportable subjects through liberalizing legal regulations and/or enforcement practices. Conformist protests do not only ignore the socio-political arrangements upholding the differentiation between citizen and aliens, they may even, to some extent, reaffirm these arrangements, by suggesting that the enforcement of deportation is unjustified for reasons that apply (exclusively) in that individual case. We speak of conformist aspirations for change if the protest carries claims to halt the enforcement in specific, exceptional cases. Protests exhibiting this dimension of change aspiration can be also categorized as consensus-oriented (cf. Diani and Bison 2004), since they do not demand political changes beyond the concession to make an exemption in a particular case.

At this point, let us emphasize that we understand anti-deportation protests as political protests, regardless of which dimension of change aspiration they present. Protests are a form of political action that seeks to influence a political decision or process, which is perceived by protesters as having negative consequences for themselves, another group or society as a whole (Rucht 2007, 708). Protests exert pressure on power holders for (or against) change, which makes them fundamentally political (Schaeffer 2014, 11). For instance, by calling for a halt of or the reversion of a deportation in an individual case, protests challenge decisions by power holders about law enforcement. So, we are not questioning the political nature of certain protests, rather we are interested in exploring the extent to which protests contest the practice of deportation.

## Findings: overt conformist and covert reformist and aspirations for change

In this section, we will apply this scheme of change aspirations to identify to which extent anti-deportation protests in Austria do challenge the system of deportation. For sure, any typology, also the one presented below, is based upon ideal types and neatly separated dimensions, whereas reality is often more messy. Actors engaged in one and the same protest event may hold different aspirations for change and protest campaigns might change over time. So, there are some limitations to applying typologies, but our approach allows us to capture important tendencies and patterns in political change aspirations that characterize anti-deportation protests. As we will present in the following, we found a dominance of “overt” conformism, and a pattern of “covert” reformism.

### Overt conformism

In total, we identified 311 protest events during the analysed period for Austria. Most of these protest events (235 in absolute numbers) took place between 2004 and 2013. This increase of protest events over time emerged despite a decrease in actual deportations. In contrast to protest events in Austria’s neighbouring countries Germany and Switzerland, the large share of these events (83%) were protests for individual deportees. The most numerous claims identified were demands to halt or prevent a specific deportation. Claims for a general legal right to stay, in turn, were relatively few and, notably, there were no demands in the analysed protest events to abolish the system of deportation. Only a few claims for reforms of deportation policies and enforcement practices emerged (see Table 2).

**Table 2.** Claims in Austria, Germany and Switzerland (1993–2013).

	AT 93-13	AT93-03	AT 04-13	DE	CH
Pro deportation	3.5%	5.1%	3%	1.4%	1.2%
Preventing a specific deportation	55.5%	50%	56.6%	25.6%	30.9%
Against deportation in general	0%	0%	0%	12.6%	3%
Reform of deportation practices/policies	21%	25.6%	19.1%	22.8%	41.8%
Reform/abolition Dublin	0.3%	1.3%	5.5%	1%	0.6%
General right to stay	4.2%	2.6%	9.4%	5.3%	13.9%
Right to stay for certain individuals	7.1%	7.7%	1.7%	26.4%	7.3%
Return of deported individuals	3.2%	7.7%	1.7%	3%	1.2%
Others	5.2%	7.7%	4.3%	1.8%	0%

Notes*:* number of protest claims in percentages (rounded off to one decimal place) of all claims in the same country; *n* = 310 claims (AT) (AT 1993-2003: *n* = 75; AT 2004-2013 *n* = 235); *n* = 495 claims (DE); *n* = 165 claims (CH).

In order to categorize protests in a systematic fashion in the PEA, we identified and coded the primary claim for each protest event. Protests could thus also entail other minor demands we did not capture. However, this does not diminish the predominance of conformist change aspirations identified. The high proportion of claims that call for a waiver of the enforcement of a specific deportation, in combination with the low number of claims for a legal right to stay, and the overall absence of claims opposing deportations as such, emphasize the dominance of conformist change aspirations in anti-deportation activities in Austria.

The focus on individual deportations is related to the group of protest actors. As the protest event analysis revealed, in addition to deportees themselves, it was mainly individuals with and without personal ties to the deportees that became active in anti-deportation protests, whereas organized activists (e.g. members of NGOs, political organizations, migrant communities or religious organizations) were significantly less engaged (see Table 3).

**Table 3.** Major protest actors in Austria per decade.

Major actors	1993–2003	2004–13
Potential deportees	36%	25%
Grass-root organizations and individuals with personal ties mentioned	8%	14%
Grass-root organizations and individuals with no personal ties mentioned	14%	23%
NGO	16%	10%
Church-related	4%	4%
Politics	8%	13%
Other	14%	12%

Given are the % of protests during the indicated time period, in which a particular kind of actor was the main actor; other type of actors may also be involved.

The fact that protesters with personal ties constitute a substantial pool of protesters shows that social relations play a relevant role in getting people involved in pro-migrant protests. In other words, in a society in which immigration is largely viewed as a problem and where protest culture is weak, it is social contacts that can function as an important trigger for siding with and protesting on behalf of immigrants (cf. also Rosenberger and Winkler 2014).

Our quantitative data set indicates that the ideological profile of protestors does play a role for the dominance of a conformist, consensus-oriented protest pattern. Usually, people who choose to protest tend to be on the left of the political spectrum (Hutter 2014), an ideological group that stands for radical change. In deportation protests in Austria for the observed period, groups were not defined by partisanship. Protesters included people from all walks of life. This feature is particularly pronounced in case mobilizations (cf. also Ruedin, Rosenberger, and Merhaut 2018).

In his seminal work on immigration in the United States, Astride R. Zolberg (2006) famously spoke of “strange bedfellows” when it comes to pro-immigrant advocates. Persons who protest on behalf of individual migrants represent a specific case of strange bedfellows. Protesters do not come together in the first place because they share a similar political ideology about immigration; they come together because they are concerned individuals, often with ties to the person(s) threatened by deportation: a local priest, a soccer coach, a neighbour (cf. Ellermann 2006, 159). Protesters are often ordinary citizens and acquaintances rather than classical activists with a shared political vision. This specific collectivity is also related to the locality of protest. The majority of anti-deportation protest events in Austria emerged on a sub-national level within local communities (cf. Merhaut and Stern 2018). Our data suggest that protests addressing national or regional political authorities entail more substantive protest demands, aimed at reforms of policies and/or enforcement practices. Local protests, in contrast, remain predominantly focused on the protection of individuals in the phase of law enforcement.

### Covert “reformism”

As we will show below, the interview data set provides us with insights into the underlying rationale and drivers of conformist protests and it reveals a kind of “covert”, reformist political change aspiration.

Protests on behalf of individual migrants, as the interview data suggest, were often initiated by feelings of compassion, personal contact and vicinity. Initially, they were not driven by a broader critique of the deportation system, policies or deportation enforcement practices. Some of our interviewees explicitly refrained from defining their engagement as an act of protest against the political authorities. Instead, protesters were keen to portray their engagement as pure manifestation of compassion rather than a form of political protest, or dissent:

[…] It was not about protesting, […].it emerged from the personal contact, getting to know them [the group of asylum seekers], and seeing their problems [… .] we would never have seen our actions as protest. We would have seen it as forms of solidarity and protection for these people. […] I did not see myself as protester […] [that’s why] I thought it was outrageous that there was a police operation to prevent us from doing anything stupid […] I know myself where the limits are, I do not need a policeman to tell me that. We never would have seen ourselves as protesters; instead this was a statement about solidarity and protection [… .] it was solidarity and compassion to acknowledge these humans and their basic needs. (Interview 1, Albertschwende case, 10 October 2015)

This conformism corresponds to the political profiles of persons engaged in these protests as highlighted above. Many of the protesters engaged in case mobilizations have not been involved in unconventional forms of political activity before. Not being an activist or an affiliated demonstrator (Klandermans et al. 2014) suggests that they were not initially guided by any strong political beliefs or ideological motives.

Lasting engagement for individual deportable persons, however, also in cases of “altruists” with no previous “political fervour” (Guigni 2001, 236) often did result in a wider dissatisfaction with restrictive policies. Respondents stressed that they had not known much about asylum and migration policies before and that their engagement had simply emerged out of getting to know the refugees and their plight. In the course of their engagement, however, “step by step the faults in the law appeared” (Interview 2, Albertschwende case, 10 October 2015) and protesters developed a consciousness that “it is the legal regulation” (Interview 2) that caused “misery” (Interview 2). Another interviewee stressed that activism shifted his/her view of the authorities from overwhelmingly positive to negative, i.e. power holders became the target of anger and discontent. In other words, grievances about injustices emerged in the wake of case mobilization as the following passage illustrates:

It [my engagement] helped me to realize that the European rules do not work properly, and it helped me to see how the state is treating these people, when they do not have a lobby, [… .] it is that which angered me the most. Damn, I was of the opinion that we are in a good republic and I had the highest, the highest regard for our authorities, but I really have lost that [opinion]. (Interview 3, Albertschwende case, 10 October 2015)

Referring to case mobilization, Anderson, Gibney, and Paoletti (2011, 559) have emphasized, “there is little evidence that seeing the realities of immigration enforcement up close necessarily changes people’s attitudes on deportation in more general policy terms”. Our findings contradict this diagnosis to some extent.

Protests on behalf of deportees, however, clearly prioritize the fate of individuals over other protest goals, which eventually reduces the extent to which these protests challenge power holders. At the same time, this prioritization does not imply that there is no aspiration for more substantive change, or, as another interviewee put it:

[…] the highest priority was that these five people could stay here, but given that it is not only five people, given that it is a big, societal problem of many thousands of people, our communication work also had a second priority, namely, that the perspective is on change, and that Europe gets an asylum system in accordance with the rule of law, which is social and dignified. (Interview 4, Albertschwende case, 10 October 2015)

Our interview data show that case mobilization often emerges out of compassion, i.e. it is initially restricted to siding with individuals. In the course of the mobilizations process, however, protesters develop grievances that exceed dissatisfaction over individual cases. Grievances arise about asylum policies and political authorities responsible for them. In other words, compassion for a few can trigger political fervour that aspires to change for many.

The analysis, however, also suggests that this political fervour is not necessarily transferred to the streets, let alone made the major focus in claim making. The quantitative data, measuring claim making in protest events supports this diagnosis. Conformism is a persistent feature in anti-deportation protests in Austria over time. Protests demanding that the deportation of individuals be waived have actually increased over time, and demands for a reform of deportation practices and policies have decreased (see Table 2). How can we understand this pervasiveness and dominance of conformism? Why do existing grievances about policies and enforcement practices remain largely covert? In the next section, we provide two explanations for this conformism.

## Drivers of conformism

First, much of these rather conformist voices are related to the composition of the protest communities. As noted before, protest groups are often composed of diverse ideological and social segments, including first-time, non-affiliated protesters. Based on the PEA of media reports, we can see that it is non-organized individuals and grassroots organizations who tend to stand up against the enforcement of deportations in individual cases. In contrast, NGOs are relatively less likely to call for case-specific solutions and instead call for policy reforms. While the number of grassroots organizations and protesting individuals increased proportionally over time, the participation of NGOs in protests decreased over the same period (see Table 3), which partly explains the pervasiveness of “conformist” protests.

Secondly, an analysis of our interview data highlights that conformism is also driven by tactical considerations of protesters. Protest actors have to consider which demands are acceptable, and therefore more likely to succeed, in a given socio-political setting. As outlined above, the Austrian context is characterized by a particularly restrictive environment regarding asylum and migration, both in the wider public and its political elites, who are the target of protest. Restricting the major focus in claim making on the protection of specific individuals instead of openly contesting the practice of deportation, and/or widely accepted migration control policies more generally, is perceived by the protesters as the more opportune choice. As Ellermann (2006, 131) argued, protests on behalf of individuals are indeed able to attract the support of elected officials and whip up public attention because they avoid the struggle for fundamental issues, focusing instead on the worthiness of particular individuals.

To be more precise, conformist/consensus-oriented claim making is rooted in a trade-off perceived by protest actors between successfully supporting migrants in need of protection now and changing restrictive policy regimes for all in the longer term. A reoccurring theme in our interviews was the perception that contesting deportation policies and the authorities publicly would potentially endanger the protection of specific individuals facing deportation. We encountered this concern in all of the five instances of case mobilizations we studied that “too much politicization” would impede the protection of individual deportees.

Some respondents also pointed towards other players, such as the media and asylum lawyers, noting that their capacity to support migrants in their struggle against deportation would also suffer if protests would openly push for substantive reforms of the deportation state:

There are also these media experts who say, we can sell it better to the media if we don’t talk about big politics, because readers areńt interested in that […] but also lawyers [supporting refugees] are in a complicated situation, because, of course, they can do their work much more easily if they can cooperate to some extent with the aliens police [… .], so for them it is a concern that they might mess up a good agreement with the authorities if they are part of a radical campaign. (Interview 5, Ehiro case, 21 September 2015)

Identifying “conformism” as part of a protest tactic, the Austrian case study links to wider debates in social movement scholarship (see Edwards 2014) on strategies, tactics and dilemmas of protesters. This finding also highlights the fact that the political opportunities that would encourage protesters to openly push for substantive change have been widely absent in the “anti-immigrant heartland” Austria.

In sum, the moderate scope of political change aimed for in anti-deportation protests and the limited extent to which protesters challenge power holders are thus partly shaped by tactical considerations. Thus, there is reason to believe that the existing strict public and political positioning towards immigration play a decisive role for “conformist protests”. At the same time, our findings also suggest that whether protest is characterized by radicalism, reformism or conformism is influenced by the ideological composition of the protesting groups and the specific local setting in which protest emerges.

## Conclusions

This article studied change aspirations within anti-deportation protests in the “anti-immigrant heartland” Austria. We developed and applied a typology of political change aspirations (transformational/radical, reformist and conformist/moderate), and we examined the scope of political change that anti-deportation protests are engaging for. This analysis is based on longitudinal data of protest events (1993–2013). Then, drawing on protest case studies, we provided insights into motives and rationales in order to understand the pervasiveness of conformist/moderate change aspirations.

We have shown that anti-deportation protests within a political environment dominated by restrictive attitudes and policies towards immigration such as Austria are predominantly case mobilizations and feature moderate change aspirations towards existing policies and power holders. In line with other studies, our analysis has emphasized that the extent to which protests are about disrupting established orders enshrined in the practice of deportation is often limited. Their focus is not on abolishing or reforming the legal framework the practice of deportation rests on, rather it tends to be on protecting specific individual deportees, i.e. on waiving the enforcement of the law in specific cases. However, in contrast to previous research on case mobilizations, which has concluded that there is little evidence that people who engage in case mobilization hold grievances about deportation in more fundamental terms, our findings actually suggest that they do. While some of the protesters involved in case mobilizations initially did not hold a politicized attitude vis-à-vis the wider societal and political context in which struggles about deportations take place (cf. Klandermans et al. 2014), grievances about injustices executed by political authorities emerged in the wake of protest engagement.

Protesters tend to hold back on acting upon these grievances, though, in order to achieve their primary goal of protecting the individuals they mobilized for in the first place. We were able to demonstrate that the lack of overt radical or even reformist change aspirations has to be partly understood as a tactic by advocacy actors. Empirically, it became obvious that protesters considered moderate claims focusing on individuals more likely to succeed than substantive claims for political change. Protesters were aware that such moderate claims would resonate more with the authorities and the wider public. Anti-deportation protests are thus powerful, not despite their ʻconformism', but to some extent because of it. In fact, case mobilization in Austria has often been successful and has produced cracks in the deportation system (see Rosenberger and Winkler 2014). They are what Antje Ellermann (2006) evocatively called “a nightmare” for bureaucrats and political decision makers, constraining the capacity of the nation state to remove people from its territory. Case mobilizations might not openly oppose the legal framework of deportation or push for an overhaul of return policies, but they certainly pose a challenge to power holders, policymakers and bureaucrats.

However, as the strict policy approach towards migration control and forced removals of the current far-right government in Austria since 2017 indicate, the persistence of the government stance is decisive for the success of protests, regardless of whether they raise transformational, reformist or a moderate claims. Put it differently: when the government runs a strict no-exception policy, advocacy groups remain powerless and cracks in the deportation machinery fail to materialize. The success of protests in protecting deportees seems to ultimately depend on institutional political power, government actors and their attitude towards both humanity and civil society.

## Notes

1 Author SR was the project leader for Austria throughout the entire project period. Author LHA was involved at the last stage of the project, analysing the research question raised in this article based upon data collected by the Taking Sides consortium.

2 The five protest cases are: Arigona Zogaj case: 2005–2010, family from Kosovo Komani case: 2010, children and their father Karrica case: 2010, family, including a boy playing football in the Austrian under-11-division Ehiro case: 2010, Nigerian student Albertschwende case: 2015, Dublin case of five Syrian refugees
